# Emerging behavioral and neuroimaging biomarkers for early and accurate characterization of autism spectrum disorders: a systematic review

**DOI:** 10.1038/s41398-020-01178-6

**Published:** 2021-01-13

**Authors:** Chandrakanta S. Hiremath, Kommu John Vijay Sagar, B. K. Yamini, Akhila S. Girimaji, Raghavendra Kumar, Sanivarapu Lakshmi Sravanti, Hansashree Padmanabha, K. N. Vykunta Raju, M. Thomas Kishore, Preeti Jacob, Jitender Saini, Rose D. Bharath, Shekhar P. Seshadri, Manoj Kumar

**Affiliations:** 1 Department of Neuroimaging and Interventional Radiology, National Institute of Mental Health and Neurosciences, Bengaluru, India; 2 Department of Child and Adolescent Psychiatry, National Institute of Mental Health and Neurosciences, Bengaluru, India; 3 Department of Speech Pathology and Audiology, National Institute of Mental Health and Neurosciences, Bengaluru, India; 4 Department of Neurology, National Institute of Mental Health and Neuroscience, Bengaluru, India; 5 Department of Pediatric Neurology, Indira Gandhi Institute of Child Health, Bengaluru, India; 6 Department of Clinical Psychology, National Institute of Mental Health and Neuroscience, Bengaluru, India

**Keywords:** Autism spectrum disorders, Diagnostic markers

## Abstract

The possibility of early treatment and a better outcome is the direct product of early identification and characterization of any pathological condition. Autism spectrum disorder (ASD) is a neurodevelopmental disorder characterized by impairment in social communication, restricted, and repetitive patterns of behavior. In recent times, various tools and methods have been developed for the early identification and characterization of ASD features as early as 6 months of age. Thorough and exhaustive research has been done to identify biomarkers in ASD using noninvasive neuroimaging and various molecular methods. By employing advanced assessment tools such as MRI and behavioral assessment methods for accurate characterization of the ASD features and may facilitate pre-emptive interventional and targeted therapy programs. However, the application of advanced quantitative MRI methods is still confined to investigational/laboratory settings, and the clinical implication of these imaging methods in personalized medicine is still in infancy. Longitudinal research studies in neurodevelopmental disorders are the need of the hour for accurate characterization of brain–behavioral changes that could be monitored over a period of time. These findings would be more reliable and consistent with translating into the clinics. This review article aims to focus on the recent advancement of early biomarkers for the characterization of ASD features at a younger age using behavioral and quantitative MRI methods.

## Introduction

### Background

Autism spectrum disorder (ASD) comprises a group of pervasive neurodevelopmental disorders characterized by impaired social communication, restricted and repetitive patterns of behaviors^1^. ASD is also a significant public health concern with a severe long-term outcome. The adverse impact of ASD is devastating and multi-faceted, and it affects not only the child but also its siblings and parents of the affected child. Due to the underlying condition, it also significantly disturbs routine function in day-to-day life of an affected family.

As established from the past studies that ASD is a very heterogeneous condition as evidenced by its varied clinical presentation^2,3^. Various studies, together with anecdotal evidence, suggest that ASD is gender bias in nature and the male to female ratio ranges from 2:1 to 8:1, one of the most consistent epidemiological findings in neurodevelopmental disorders^4–6^. Leo Kanner conducted a study on a small group of children with ASD, and reported that the incidence rate was four times higher in the boys compared to the girls^7^. This male preponderance is almost the same across several other neurodevelopmental disorders, including ADHD^8^ and intellectual disability^9^. It has led to speculations that a shared mechanism might underlie this gender-specific increased vulnerability and its association with copy number variations (CNVs)^10^ related to variation in genetic architecture in various neurodevelopmental and neuropsychiatric disorders. These CNVs play a very crucial role in the genetic architecture of neurodevelopmental disorders and various genomic hemi-deletions or hemi-duplications have been also consistently reported in individuals affected with ASD^2–5^ and ADHD. The study of variability in the phenotypic expression implicates the presence of secondary CNVs and genetic background of the family^11^. A gender-specific variation in certain CNVs was reported, like 8:1 male:female ratio among individuals with ASD carrying the 22q11.2 duplication, and 1.3:1 male:female ratio in those carrying the 16p11.2 deletion^12^. There are several preclinical studies in animal models which also demonstrates an association between the CNVs in autism. This gender bias is highly influenced by the presence of ASD-associated risk factors such as specific comorbidities, specific CNVs, mutational burden, and pre-existing family history and these factors contribute to the variability in symptom presentation in individuals with CNVs in 16p11.2 gene^11,13,14^. Recent studies have also demonstrated the relationship between structural brain changes with molecular genetic variation in CNVs in ASD^12,15^. Several molecular genetic studies from ASD populations have also reported the genetic alterations in multiple genes in ASD^16–18^.

Magnetic resonance imaging (MRI) is a noninvasive imaging method, which produces three-dimensional detailed internal anatomical images of various structures, tissue, and organs. MRI methods played a pivotal role in providing in vivo information in a noninvasive manner at a younger age to identify aberration in brain development that can help in early identification, prediction, and correlates with behavioral symptoms to characterize the ASD features^19,20^. This valuable information will be of an enormous help in the planning of targeted early behavioral, language, and interventional therapies at younger age to provide a better clinical outcomes^21^. It is known that implementing pre-emptive interventional therapies, such as applied behavioral analysis have a profound effect, and have a high chances of long-term improvement as younger age demonstrate more brain functional plasticity compare to an older age^22^.

MRI methods can provide both qualitative and quantitative information and have been used routinely in clinical practice. MRI based approaches have been used to study the morphological, volumetric, metabolic, structural, and functional brain changes in ASD and various other pathological conditions. The volumetric MRI findings in ASD children between 2 and 5 years of age revealed regional developmental abnormalities in frontal and temporal lobes in ASD cohort compared to healthy controls. Reduction in amygdala volume as well as global gray and white matter tissue are the most common finding reported in ASD group compared to age-matched healthy controls.

Diffusion tensor imaging (DTI) is another quantitative MRI method, which measures the microscopic movement of water diffusion in the brain and is very sensitive in providing the information on the microstructural integrity of the tissue, orientation, and the connectivity of the white matter fibers in the brain^23–28^. This method is widely applied to investigate the microstructural tissue integrity and structural connectivity of the brain regions based on the molecular diffusion of water in a tissue in various pathological conditions, including ASD^29,30^. The most commonly used DTI metrics are fractional anisotropy (FA), which quantifies the strength of preferential direction of molecular diffusion of water along the white matter tracts, and the mean diffusivity (MD), which measures the magnitude of diffusion of water molecules in a tissue. DTI along with behavioral and cognitive measures have been shown useful in elucidating the relationship between the integrity of white matter pathways and the efficiency of cognitive and neural processing during brain development^31^.

Similarly, the functional MRI (fMRI), which detects the blood oxygen level-dependent (BOLD) changes in MRI signal that arise when changes in neuronal activity occur with a change in brain state. An external stimulus or task^32^ can also generate this signal change. The fMRI produces images related to neuronal activity of the brain, which provides a piece of unique and valuable information for applications in clinical neurosciences, basic and translational^33^.

Various assessment tools include screening and diagnostic method to characterize the ASD features at a very young age and these methods can also be applied to measure the severity of the ASD. Several developmental assessment tools are used to identify how children with ASD differ from typically developing children in various domains of brain development such as cognition, receptive and expressive language, gross and fine motor skills, etc. (Table 1). The description of few important assessment tools are as follows:

1. Modified Checklist for Autism Toddlers-Revised/Follow-up (M-CHAT-R/F)^34^ is a widely used screening tool comprising of 20 items and gives the information regarding whether the child is at no risk, medium, or high risk for ASD.
2. Childhood Autism Rating Scale (CARS)^35^ is a behavior rating scale consisting of two 15-item rating scales and an unscored parent/caregiver questionnaire, which assists in making ASD diagnosis and knowing the severity.
3. Autism Diagnostic Observation Schedule (ADOS)^36^ and Autism Diagnostic Interview-Revised (ADI-R)^37^ are also diagnostic tools. ADOS is a task-based tool consisting of four modules wherein depending on the child’s ability, suitable module is used. ADOS-2 was published in 2012, and has updated norms, improved algorithms for modules 1–3, and have included a new toddler module that facilitates assessment in children ages between 12 and 30 months.
4. ADI-R is a standardized parent interview-based questionnaire that is useful in distinguishing ASD features from other developmental disorders and helps in further planning the treatment. The ADI-R and ADOS stood out with the largest evidence base and also with a highest sensitivity and specificity. When the ADI-R and ADOS were used in a combination they revealed levels of accuracy very similar to the correct classification rates for the current ‘*gold standard*’ diagnostic procedure viz. 80.8% for ASD^38^.
5. Mullen Scale of Early Learning (MSEL)^39^ is a developmental assessment tool used as a measure of cognitive development comprising fine and gross motor skills, visual reception, expressive and receptive language. For young children, this early learning composite score is considered equivalent to a more traditional “*IQ*” score or a developmental standard score.
6. Social Communication Questionnaire (SCQ)^40^ is widely used as a screener and was designed as a questionnaire version of the ADI-R. It has two versions: lifetime and current in yes/no format of the questionnaire.

**Table 1:** Developmental and behavioral assessment tools used to characterize the features of children with autism.

Assessment tools	Age	Description
Modified Checklist for Autism Toddlers-Revised/Follow-up (M-CHAT–R/F)^109^	16–30 months	It is a two-stage parent-report screening tool. Items 2, 5, and 12 are critical if they are answered ‘yes,’. When the rest of the items are answered ‘no,’ the risk for ASD is high.
Childhood Autism Rating Scale (CARS)^110,111^	Two years and above	The diagnostic tool consists of 14 domains assessing behaviors associated with ASD, with a 15th domain rating general impressions of ASD. Each domain is scored on a scale ranging from one to four; higher scores are associated with a higher level of impairment.
Autism Diagnostic Observation Schedule (ADOS)^36^	12 months through adulthood	Consist of a series of structured and semi-structured tasks that involve social interaction between the examiner and the person under assessment. This assessment comprises of four modules differing in developmental and language levels, ranging from nonverbal to verbally-fluent.
Autism Diagnostic Inventory-Revised (ADI-R)^37^	The mental age of about 18 months to adulthood	It is a standardized semi-structured clinical interview for caregivers of children and adults. The interview contains 93 items and focuses on behavior in three content areas: quality of social interaction; communication and language and repetitive, restricted, and stereotyped interests and behavior.
Mullen Scale of Early Learning (MSEL)^39,112^.	Two days to 69 months	It is an assessment battery designed to measure development in infants and preschoolers. 124 items measure specific domains, including gross motor functions, visual reception, fine motor, and receptive and expressive language skills.
Social Communication Questionnaire (SCQ)^40^	The mental age of 2 years and above	It is 40 items, yes or no, parent-report screening measure mainly focuses on the items relating to ASD symptomology primarily observed by a caregiver.

Therefore, various tools are used to screen for risk and severity of the ASD, distinguishing from other neurodevelopmental disorders, treatment planning, and as well as for research purposes. Currently, there is no standard method of care or medications available for ASD. However, an early detection and pre-emptive behavioral-based intervention can make a significant difference in clinical management, as well as an overall improvement in the quality of life of children with ASD. Therefore, early detection before clinical symptoms appear is of paramount importance for clinical management and in treatment of ASD.

Considering the potential application of early assessment of neurodevelopmental and behavioral characteristics by using and neuroimaging methods in neurodevelopmental disorders, the main objective of this review article is to discuss the brain–behavior abnormalities using noninvasive MR imaging methods along with neurodevelopmental and behavioral assessment tools to characterize the ASD features at early stage. It is widely reported that neuroimaging methods play a crucial role in identifying the noninvasive imaging biomarkers for early diagnosis, and accurate characterization leading to start pre-emptive interventional therapies in ASD.

### Clinical and behavioral characteristics of ASD

Neurodevelopmental disorders typically manifest early in the developmental stage, most often before the child enters grade school; hence diagnosis and treatment of the neurodevelopmental disorders can be difficult. There are no specific tests that can predict developmental and cognitive deficits before early childhood in various neurodevelopmental disorders, including ASD.

Several characteristic features have been reported in ASD for early characterization of the disorders and commonly referred to as “*Red flags for the ASD*^”^^41^. Various characteristic features have been reported at the various time point of the age, like no eye contact by 6 months of age, no response to name-calling, and no social referencing by the age of 10 months, no imitation and two meaningful words by 12 months of the age, no proto-declarative and proto-imperative pointing by 14 months of the age, and no joint attention by 18 months of age. Infants at risk for and later diagnosed with ASD showed a decline (from previous normative levels) in eye fixation within the first 2–6 months of age^42^. This pattern was not observed in typically developing infants. Children with autism showed lower rates of canonical babbling and fewer speech-like vocalizations across the age, i.e., 6–24 months of age (based on early home videos) than did typically developing peers^43^.

ASD symptoms and features typically manifest around 2 years of age, and most of the children with ASD prefer solitary play; no pretend or symbolic play by 2 years of age^1^, and cooperative play by 3 years of the age. Children with ASD interact only on a need basis and demonstrate the restricted patterns of behavior, including focus only on a particular part of the toy rather than playing with a whole toy, indicating narrow interests^1^, a repetitive behavior includes hand flapping, head rocking, or toe walking^1^.

Due to heterogeneity, children with ASD either have hypersensitive or hyposensitive characteristic features. Children with ASD respond to a stimulus either by excessive touching, smelling, and mouthing or do not like to touch particular texture or sticky things^1^. Self-injurious behaviors like head banging and aggressiveness can also be seen most commonly in children with ASD. Often time it has also been noted that some children with ASD might also demonstrate the normal development up to a certain age and begin to regress development just before or around 2 years of the age^44^. The first epidemiological study on ASD by Victor Lotter in 1966 put the number of those affected at 4.5 per 10,000^45^. However, the current information available on the center for disease control (CDC) prevention has significantly change, and the current prevalence rate of ASD is 1 in 54 children^46^.

### Aetiology

ASD aetiology remains elusive and multifactorial. There are many hypotheses and known risk factors that contribute to developing ASD features. However, there is no single known cause for ASD and pathophysiology of the disease significantly remains unclear. Though, it is unequivocally accepted that the genetic and environmental factors thought to play a significant role in ASD. Abnormal neural connections, volumetric brain abnormalities, and altered structural, and functional brain connectivity lead to the most profound socio-behavioral abnormalities in ASD^47^.

Aberrant brain growth with disturbed regional growth trajectories plays a major role in the characterization of ASD. Several studies have reported alterations in lifetime trajectories of several brain regions^47–50^. There are various other risk factors identified and known to be associated with the prevalence of ASD, such as older siblings with ASD^51^, parental age^21^, prematurity, and multiple other prenatal, perinatal, and neonatal adversities^20^. Volk et al. have also identified that exposure to harmful chemicals such as lead, mercury, and pollution during pregnancy may also contribute to developing ASD^52^.

Besides the risk factors, many researchers have also identified other biomarkers for ASD by conducting investigations from blood samples or using noninvasive MRI methods to understand the pathophysiology of the disorder in a better way. However, the progress made in identifying the diagnostic biomarkers is still in infancy, and findings reported from individual studies are confined to research purposes only. Translating these findings into clinical settings is still a challenge. After generalizing these research findings, there will be a significant advancement in early and accurate diagnosis, characterization, and clinical management of ASD patients at an early stage. It will also significantly reduce the age of ASD diagnosis^53^. There will be no need for the clinician to wait until 3 years of age to make a clinical diagnosis of ASD based on behavioral and developmental observations.

## Methods

### Literature search strategy

The following strategies were used to identify appropriate neuroimaging, developmental, and behavioral studies specific to ASD databases such as PubMed, Google Scholar, Scopus, Ovid Medline, and Cochrane databases to retrieve the relevant studies published between January 2005 and May 2020. To identify the appropriate studies during the literature search, we used the keywords, terms such as “imaging biomarkers”, “behavioral assessment”, “Autism and Neurodevelopmental disorder”, and “Autism”. The review article describing specific behavioral and imaging methods in characterizing the ASD features were reviewed to determine if specific biomarkers had been developed for the specific research areas.

### Study selection

Studies were included in this review article if they met specific criteria, as mentioned below.

1. The included studies were:
  A. Confirmation of ASD using DSM-5 at the time of testing.
  B. Age ranges between 0 and 6 years.
  C. Use of validated behavioral and MR imaging methods in the study.
  D. Human randomized controlled trials, non-randomized trials, case studies, and case series reported direct clinical imaging findings as a characteristic feature of autism.
2. The following exclusion criteria were also applied:
  A. The studies with no MR imaging methods.
  B. Above 6 years of age.
  C. Studies with other neurodevelopmental conditions like attention deficit hyperactivity disorder (ADHD) and intellectual developmental disorders (IDD).
  D. Studies with only genetic analysis.

After reviewing all the defined studies from the collected literature, the studies with promising findings were included in this review article. We included the studies in which developmental and behavioral assessment and MR imaging methods were used to characterize the ASD features. The MRI studies have included one or more of the following MRI methods, i.e., conventional and advanced MRI methods. The advanced MRI (DTI and fMRI) methods provide quantitative information on volumetric, structural, and functional connectivity differences in the brain between ASD and typically developing children. Few studies have also reported a correlation between quantitative MRI with behavioral and developmental parameters, which are discussed here.

## Discussion

### Neuroimaging studies in ASD

Brain development throughout the life span is a complex and dynamic process that should be considered early infancy, even prenatally^54^. Recently, the rapid development of noninvasive brain imaging technology, a new generation of imaging technique such as MRI, holds great promise for its ability to reveal structural and functional brain alterations during development in infants, children, and adolescents (Table 2). Given its noninvasive nature, MRI could be used in clinical practice as part of the comprehensive clinical assessment of ASD patients to exclude brain alterations. As the relationship between the postnatal CNS structural development and functional capacities of children from birth to adolescence gradually unfolds, the potential for identifying early delays in cognitive development mounting effective treatment and prevention strategies increases^55^. Thus, it allows pre-emptive interventional resources and biological-based therapies for targeted intervention to reduce the frequent burden of intellectual disabilities in the ASD population. Although studies using structural and functional MRI have highlighted alterations in the neuroanatomy of ASD in the past few decades^56–58^, a univocal, reliable, and consistent pattern of alterations is yet to be identified. Also, there is not enough information available on imaging perspective to clearly understand the underlying pathophysiology of ASD at a younger age, i.e., below 6 years of age.

**Table 2:** Summary of the behavioral and imaging findings in ASD.

Reference	Study type	Subjects	Age range	Method	Brain regions	Findings
Hazlett et al.^64^	A prospective and longitudinal study	High and low-risk ASD	6–24 months	Structural brain MRI	Cortical surface area	Hyperexpansion of cortical surface area precedes brain volume overgrowth
Nordahl et al.^68^	A prospective and longitudinal study	ASD and typically developing	2–4 years	Structural brain MRI	Amygdala	Increased total cerebral and amygdala volume
Pote et al.^65^	A prospective and longitudinal study	High and low-risk ASD	4–36 months	Structural brain MRI	Cerebellum and subcortical regions	Larger cerebellum and subcortical volumes
Schumann et al.^71^	A prospective and longitudinal study	ASD and typically developing	1.5–5 years	Structural brain MRI	Cerebral gray and white matter	Increase in cerebral gray and white matter specifically in frontal, temporal, and cingulate cortices
Shen et al.^66,67^	A prospective and longitudinal study	High-risk and low-risk ASD	6 months to 3 years	Structural brain MRI	Extra-axial cerebrospinal fluid	Increase in volume of extra-axial cerebrospinal fluid
Zhu et al.^69^	Prospective and cross-sectional	ASD and typically developing	2–5 years	Structural brain MRI	Amygdala	Larger bilateral amygdala volumes
Conti et al.^97^	Prospective and cross-sectional	ASD and other developmental delays	Below 36 months	DTI	White matter connectivity	The over-connectivity pattern in ASD occurred in networks involving the frontotemporal nodes and basal ganglia
Fingher et al.^86^	Prospective and cross-sectional	ASD and typically developing	<2.5 years	DTI	Corpus callosum	Fractional anisotropy and the cross-sectional area of the temporal corpus callosum tract were significantly larger
Solso et al.^94^	Prospective and longitudinal	ASD and typically developing	12–48 months	DTI	Frontal lobe	Frontal fiber tracts displayed deviant early development and age-related changes
Weinstein et al.^84^	Prospective and cross-sectional	ASD and typically developing	1.5–5.8 years	DTI	Corpus callosum left superior longitudinal fasciculus and right and left cingulum	Increase in fractional anisotropy was seen in corpus callosum as well as in the left cingulum
Wolff et al.^88^	A prospective and longitudinal study	High risk and ASD	6–24 months	DTI	White matter fiber tract	Aberrant development of WM pathways may precede the manifestation of autistic symptoms in the first year of life
Wolff et al.^87^	A prospective and longitudinal study	High risk and ASD	6–24 months	DTI	Cerebellum and corpus callosum	Aberrant structural properties of callosal and cerebellar white matter pathways were observed
Xiao et al.^98^	Prospective and cross-sectional	ASD and developmental delay	2–3 years	Structural imaging and DTI	Gray and white matter volumes and corpus callosum, cingulate cortex, and limbic lobes	An increase in global gray and white matter volume and higher fractional anisotropy value was also observed in the corpus callosum, posterior cingulate cortex, and limbic lobes
Ciarrusta et al.^113^	Prospective and cross-sectional	High risk and typically developing	Neonates	Resting-state fMRI	Resting-state networks	Significantly high regional homogeneity within multiple resting-state networks
Eggebrecht et al.^103^	Prospective and longitudinal	High risk and typically developing	12–24 months	fMRI	visual, dorsal attention and default mode network	Association between joint attention and these mentioned networks were found
Eyler et al.^105^	Prospective and longitudinal	ASD and typically developing	12–48 months	Resting-state fMRI	Temporal	Abnormally right-lateralized temporal cortex response to language
McKinnon et al.^106^	Prospective and longitudinal	High risk and typically developing	12–24 months	Resting-state fMRI	Dorsal attention, subcortical, and default mode network	Association between these networks and RRBs were found except that for self-injurious behaviors

### Volumetric changes in ASD

Size of the brain is often measured by weight, sometimes by volume (via MRI scans or by skull volume), and referred as cranial capacity. The changes in brain volume differ depending on several factors, such as age, environment, and body size. Mostly, the brain volume is measured in a cubic centimeter, and an average volume of a modern human brain is between 1300 and 1500 cm^3^. Any deviation in brain volume results in structural changes in the brain tissue and might alter behavioral or functional patterns or vice versa. Studies on structural and volumetric changes in the ASD explored their relationship with behavioral changes. Findings from some of the recent studies in ASD using structural brain MRI are summarized below.

Infants with older ASD siblings are said to be at risk of developing ASD and other related neurodevelopmental issues, more specifically with male sibling^59,60^. A prospective neuroimaging study conducted by Hazlett et al. on infants at high risk for autism reported the hyperexpansion of cortical surface area between 6 and 12 months of age followed by an increase in brain volume between 12 and 24 months. Brain volume was associated with the emergence and severity of social deficits in ASD^61^.

Pote et al. have performed a study on infants from high-risk populations and healthy controls using MRI. They observed that high-risk infants at ages 4–6 months had larger cerebellar and subcortical volumes than age-matched healthy controls. These findings were subsequently linked to repetitive behavior at 36 months of age^62^. A significantly high extra-axial fluid was observed in a longitudinal study (6–9, 12–15, and 18–24 months) at 6–9 months of age. It remained elevated in follow-up at 12–15 and 18–24 months of age in children diagnosed with ASD at 24 months of age. This extra-axial fluid is characterized by excessive CSF in subarachnoid space, specifically in the frontal lobe. The detection of elevated extra-axial fluid as early as 6 months of age is predictive of the severity of the ASD symptoms at the time of outcome measurement, i.e., between 2.5 and 3 years of age^63^. These investigators have recently replicated their previous findings and reported that the increased extra-axial CSF volume, detected by using conventional structural MRI from infancy through up to 3 years of age. Authors have suggested that the increased extra-axial CSF volume could be an early stratification biomarker of a biological-based subtype of ASD, which share the common underlying pathophysiology of the ASD^64^.

Hippocampus and amygdala are part of the limbic system in the brain. Hippocampus is responsible for memory; and amygdala for the regulation of emotions. The amygdala is relatively enlarged in ASD compared to healthy controls by 2 years of age while demonstrating a growth regression in magnitude between 2 and 4 years of age^65^. The amygdala volume in 2–5-year-old pre-school children with ASD has reported significantly larger than age-matched typically developing children. These volumetric changes were associated with behavioral and social deficits in ASD^66^. A prospective longitudinal study reported an enlarged amygdala and total cerebral volumes with the same magnitude in ASD compared to healthy controls. However, when adjusted for total cerebral volumes, amygdalar enlargement remained significant at both time points after a 1-year interval^67^.

It has been well documented through the cross-sectional and longitudinal MRI studies that the brain changes in ASD undergo an abnormal growth trajectory, which includes a period of early overgrowth followed by regression^68,69^. A longitudinal study in ASD demonstrates the regional changes in growth trajectory with significantly increased cerebral gray and white matter tissue in toddlers with ASD. The most severe spur occurs in frontal, temporal, and cingulate cortices^70^.

After considering the age and gender effect on the regional changes in abnormal growth trajectory from various regions of the brain with a quadratic age effect, mainly characterized this abnormal growth rate, except for the occipital gray matter. The gender influence significantly demonstrated that females with ASD features unveiled a more enunciated abnormal growth profile in several brain regions than males^71^.

Various studies have been reported abnormality in brain volume in ASD^72,73^. However, these findings are very heterogeneous and inconsistent. Study from Courchesne et al. have reported smaller brain at birth in ASD compared to age-matched healthy cohort and in subsequent years overgrowth occurs in ASD followed by decline/regression in brain growth^48^. Authors have speculated that excess neuron numbers may be one possible cause of early brain overgrowth and produce defects in neural patterning and wiring with aberrant exuberant long and short-distance cortical interactions impeding the functions^48^. Several studies suggest that an increase in brain volume is not a robust biomarker in ASD^74–76^. Findings, like an increase in extra-axial fluid and regional enlargement in the limbic system, still need further evaluation to replicate the findings. Also, no consensus has been reached on the nature and clinical relevance of these changes observed in the ASD population than typically developing children.

### Structural brain connectivity changes in ASD

DTI is a valuable quantitative MRI method that can measure the diffusivity of water molecules in the tissue and provide in vivo information on the microstructural integrity of the tissue^77^. The most commonly used DTI parameters are fractional anisotropy (FA), which describes the degree of directional dependence; mean diffusivity (MD), which expresses the magnitude of the diffusion^78^; axial diffusivity (AD), which represents water diffusivity parallel to the axonal fibers; and radial diffusivity (RD), which represents water diffusivity perpendicular to the axonal fibers^78,79^.

A widely accepted hypothesis regarding the findings of structural brain abnormalities in ASD is local over-connectivity and long-distance underconnectivity in various white matter pathways^80,81^. An altered brain connectivity may be a key pathophysiological feature of ASD and underlie the abnormal brain development in children with ASD at early age (pre-school)^82^. Higher FA value was observed in the corpus callosum, posterior cingulate cortex, and limbic lobes in ASD compared to healthy control^83^. Abnormal structural brain connectivity has also been reported between corpus callosum/cingulum and temporal lobes involving the inferior longitudinal fasciculus/inferior frontal-occipital fasciculus and superior longitudinal fasciculus in ASD^84^. Aberrant neural connectivity measured the structural integrity and suggested an association with abnormal social perception and cognition in ASD^85^. Abnormal FA values and altered developmental growth trajectories have been reported in 12 out of 15 white fiber tracts in infants developed ASD compared to typically developing infants. Development for most fiber tracts in infants with ASD was characterized by elevated FA at 6 months, followed by slower developmental change over time relative to infants without ASD. Thus, by 24 months of age, lower FA values were evident for those with ASD^86^. The aberrant white matter development may precede the manifestation of ASD features in the first year of life and demonstrate abnormal neural circuitry at 6 months of age-associated with restricted and repetitive behavior^87^.

In contrast, abnormal sensory responsiveness contributes to ASD diagnosis at 2 years of age^88^. These findings are also associated with abnormal structural properties of Cerebro-callosal pathways measured during infancy and toddlerhood^88^. This study further investigated the concurrent occurrence of repetitive and unusual sensory response patterns, indicated common brain–behavior relationships in ASD^88^. Reduced FA and elevated MD values in white matter tracts from various regions of the brain have been consistently reported in ASD^89–93^.

Frontal lobe white matter fiber tracts showed atypical early developmental and age-related changes, which could underlie the weakening brain connection and functioning and ultimately influence the socio-communicative behaviors in ASD^94^. Comparing with mental age-matched control subjects, the ASD group demonstrates decreased activity in an extended neural network of the brain regions enrolled in typical early language acquisition^95^. Similarly, the ASD participants demonstrated greater activation in the regions primarily within the right and medial frontal lobes of the brain compared to chronicle age-matched healthy control^47^. Laterality analyses revealed a trend toward higher enrolment of the right hemisphere in the ASD group, while left hemisphere in the chronically age-matched healthy control during the forward speech condition^96^. The correlation analysis revealed a significant positive relationship between right hemisphere frontal and temporal lobe activity for forwarding speech and receptive language skills in children with ASD. The over-connectivity pattern in ASD also occurred in networks primarily involving the frontotemporal nodes. It is known to be of utmost importance for social-skill development, and basal ganglia for restricted and repetitive behaviors^97^. Further, the non-divergent findings of structural and white matter abnormalities in ASD suggest that modifications in neural-anatomy of distinct brain regions may be involved in behavioral and cognitive deficits correlated with ASD, especially in an early age of 2–3-year-old toddlers^98^.

### Functional brain connectivity findings in ASD

The fMRI depicts changes in deoxyhemoglobin concentration consequent to the task-induced or spontaneous modulation of neural metabolism^99^. Since its inception in 1990, the method has been widely employed in thousands of neurocognition studies for clinical applications, surgical planning, monitoring treatment response, and assess the clinical outcomes. This method has also been used as a noninvasive imaging biomarker in pharmacologic studies and training programs^100^. The local underconnectivity in dorsal posterior cingulate cortex, and right medial paracentral lobule has been well-documented using fMRI studies in the ASD population^101^.

Failure in initiating joint attention is one of the early behavioral markers to characterize the ASD features and can provide a foundation for developing social communication^102^. Research by Eggebrecht et al. showed associations between specific networks like visual network and dorsal attention network, visual network, and posterior cingulate aspects of the default mode network with and initiating joint attention by doing resting-state fMRI in high-risk infants and toddlers^103^.

Failure to develop language comprehension is also considered to be one of the early warning signs of ASD^103,104^. It has been reported that children at-risk for ASD demonstrate deficit in left-hemispheric responses to speech sounds and abnormal right-lateralized temporal cortex response to language; this defect worsens with age and becomes most severe between 3 and 4 years of the age^105^. McKinnon et al. reported the restricted and repetitive behaviors and resting-state fMRI findings in high-risk ASD^106^. Furthermore, at 2 years of age, stereotyped behavior was strongly associated with more positive functional connectivity between dorsal attention and subcortical networks^101^. In contrast, restricted behavior was strongly associated with functional connectivity between default mode and dorsal attentional networks. However, no significant network-level associations were observed for self-injurious behavior^106^.

There are other modalities such as eye-tracking, positron emission tomography (PET), MR spectroscopy (MRS), and other molecular imaging methods which are also being used to characterize the ASD features. However, these methods are not discussed here as above mentioned topics are beyond the scope of this review article.

### Current challenges

There are several challenges that researchers are facing to conduct the study on younger (infants and toddlers) population, like in recruitment and assessment in general. However, these challenges become even more tedious when imaging is done on these young population. In general public, there is a stigma attached to mental health; even among the educated society, mental health literacy is low; lack of awareness about neurodevelopmental conditions that children may develop^107^ and misconceptions and fear associated with undergoing MRI scan at a younger age is huge. Administering an MRI scan on such a young child under natural sleep is the most tedious task^108^. Age-specific objective may impose additional time constraints in the recruitment process. Many of the studies are published on ASD with fewer subjects, and there is a dearth of reliable biomarkers as the findings are not yet applicable in routine clinical practice. At present, several limitations obstruct to expand our understanding on the exact pathophysiology of the disease progression. As ASD is a heterogeneous disorder, most of the studies were conducted on small sample sizes and cross-sectional. Due to heterogeneity most of the published findings are not consistent and cannot be generalized. The findings opined in this review mainly emphasized on the behavioral abnormalities associated with volumetric, structural, and functional brain connectivity in ASD using MRI.

### Future directions

Socioeconomic status and parental education play a very significant role in the early detection of features of ASD. Parents from low economic strata mostly may not be able to recognize or most often ignore the features thinking that this will improve as the child’s age progress. When parents are psycho-educated on the neurodevelopmental issue, many parents do not accept it initially even though they recognize that the child has some developmental issue and willing to start interventional therapies. However, most of them face the primary issue with accessibility and affordability of the medical services, which further delays the outcome of intervention programs. Hence, availability of trained child psychiatry personnel is another paramount component and required an urgent attention to fill the gap. These neurodevelopmental disorders demonstrate variable clinical features at various age. Hence, conducting longitudinal research studies are need of the hour to reach a consensus on the current prevalence rate and better understanding on the pathophysiology of the disease progression over the time. The longitudinal studies will also help in developing reliable imaging biomarkers by replicating the studies on a larger cohort.

## Conclusion

The imaging findings play a pivotal role in the early detection and accurate characterization of brain abnormalities and subsequent assessment of the risk of developing autism. The risk of developing ASD can be predicted and assessed by using advanced MR imaging methods, which can guide the treating physicians to plan an appropriate targeted interventional strategy to enhance the long-term clinical outcome.
